# Variation in CBC-Derived Inflammatory Biomarkers Across Histologic Subtypes of Lung Cancer: Can Histology Guide Clinical Management?

**DOI:** 10.3390/diagnostics15111437

**Published:** 2025-06-05

**Authors:** Claudia Raluca Mariean, Oana Mirela Tiuca, Alexandru Mariean, Ovidiu Simion Cotoi

**Affiliations:** 1 Doctoral School of Medicine and Pharmacy, George Emil Palade University of Medicine, Pharmacy, Science and Technology of Targu Mureș, 540142 Targu Mureș, Romania; 2 Pathophysiology Department, George Emil Palade University of Medicine, Pharmacy, Science and Technology of Targu Mureș, 540142 Targu Mureș, Romania; 3 Department of Radiology, Targu Mureș County Emergency Hospital, 540136 Targu Mureș, Romania; 4 Dermatology Department, George Emil Palade University of Medicine, Pharmacy, Science and Technology of Targu Mureș, 540142 Targu Mureș, Romania; 5 Dermatology Clinic, Mures Clinical County Hospital, 540342 Targu Mureș, Romania; 6 Pulmonology Clinic, Mures Clinical County Hospital, 540103 Targu Mureș, Romania; 7 Pathology Department, Mures Clinical County Hospital, 540011 Targu Mureș, Romania

**Keywords:** lung cancer, histologic subtypes, systemic inflammation, CBC-derived biomarkers, smoking status, COPD

## Abstract

**Background/Objectives**: The early detection of high levels of CBC-derived inflammatory biomarkers and cellular lines, as well as their variations across different histological subtypes of lung cancer, may aid in the early identification of high-risk lung cancer patients and further guide their clinical approach. **Methods**: A retrospective descriptive study was conducted and included 202 patients diagnosed with lung carcinoma at the Clinical County Hospital Mureș. The main analyzed parameters were the histological subtype and the stage of the tumor at diagnosis, white blood cell counts, and platelet counts, as well as nine CBC-derived inflammatory indexes like neutrophil-to-lymphocyte ratio (NLR), derived neutrophil-to-lymphocyte ratio (d-NLR), monocyte-to-lymphocyte ratio (MLR), platelet-to-lymphocyte ratio (PLR), eosinophil-to-neutrophil ratio (ENR), eosinophil-to-monocyte ratio (EMR), systemic inflammatory index (SII), systemic inflammatory response index (SIRI), and aggregate index of systemic inflammation (AISI). The statistical analysis was performed using the MedCalc software, version 23.0.2. Logarithmic ANOVA was used to compare groups. Normality was tested using the Shapiro–Wilk test. The Chi-square test compared categorical variables, while the independent Mann-Whitney test was used for continuous variables. **Results**: The inflammatory response increased as disease severity progressed, with NSCLC-NOS being the histological subtype with the most numerous patients outside the normal ranges. Eosinophil count differed significantly across the histologic subtypes of NSCLC, with adenocarcinoma and adenosquamous patients exhibiting the highest values. In adenocarcinoma patients, we observed that NLR and MLR levels increased progressively as the tumor stage advanced. Based on severity, differences were observed across the histological subtypes of lung cancer in stage III patients for ENR, EMR, AISI, eosinophil count, and platelet count, as well as in stage IV patients for AISI, SIRI, and SII. Disease severity impacts the associated inflammatory response in all histologic subtypes of lung cancer to varying degrees. **Conclusions**: Histological subtype might have a decisive role in shaping the systemic inflammatory profile of lung cancer patients. CBC-derived indices serve as accessible, cost-effective biomarkers for early risk assessment, aiding in the prognosis evaluation and monitoring of therapeutic response. Future studies are needed to further evaluate the histology-specific inflammatory profiles as adjunctive tools in precision oncology.

## 1. Introduction

Cancer represents a leading cause of death worldwide, being the first or the second cause of mortality before the age of 70 in 112 of 183 countries, according to the World Health Organization (WHO). Lung cancer is the second most commonly diagnosed malignancy and accounts for the highest mortality rate, as 18% of all cancer deaths are directly caused by lung carcinoma [1]. It is vital to increase efforts to understand this pathology’s complexity. Therefore, this study investigates how CBC-derived inflammatory biomarkers vary by histologic subtype and stage in lung cancer patients, aiming to identify clinically useful patterns.

Inflammation is essential in carcinogenesis, as “tumor-promoting inflammation” is a hallmark of neoplastic processes [2]. The inflammatory response is based on a precise network of many cellular types. Neutrophils, macrophages, monocytes, lymphocytes, and various granulocytes are key cells involved in the inflammatory changes associated with malignancies. Recent studies have also identified platelets as key cells in the inflammatory response and multiple processes, including atherosclerosis, infections, and neoplastic diseases [3,4]. The tumor microenvironment (TME) plays a crucial role in carcinogenesis. It comprises multiple cellular and non-cellular components that can influence tumor progression and response to therapeutic agents [2,5,6]. Complex interactions are observed in the TME between inflammatory and cancer cells. Most of the cells found in the TME are cancer cells, endothelial cells, pericytes, immune cells, and cancer-associated fibroblasts (CAFs). CAFs promote the development of an immunosuppressive environment and enhance cancer cells’ angiogenesis, proliferation, and survival [5,7]. Endothelial cells promote angiogenesis, the infiltration of immune cells, tumor progression, and metastasis [5]. The activity of immune cells plays a crucial role in tumor-associated inflammation. Leukocytes, CD4^+^ T-cells, CD8^+^ T-cells, natural killer (NK) cells, and macrophages are all key cells involved in tumor progression [8,9]. The immune components of the TME influence the response to therapy, as variations in the organization and density of these immune cellular lines may be associated with cancer prognosis. In addition, a precise characterization of the associated inflammatory changes can guide and personalize immunotherapeutic strategies used for cancer patients, with novel discoveries such as immune checkpoint inhibitors, cytokine therapy, cellular therapy, and therapeutic vaccines cited [10].

The systemic inflammatory response reflects the TME; therefore, identifying easily accessible biomarkers that depict the degree of associated inflammation represents a key direction for improving the clinical outcomes of cancer patients. The presence of inflammatory cells in the TME can influence tumor progression and impact the systemic inflammatory response in cancer patients [11].

In recent years, significant efforts have been made to identify easily accessible biomarkers that assess the immune response of cancer patients and identify patients at high risk for developing complications. Multiple parameters, including anthropometric measurements, blood tests, and imaging modalities, have been identified as potential biomarkers for tumor-associated inflammation [12,13,14,15,16].

CBC-derived inflammatory markers, such as NLR, d-NLR, EMR, ENR, PLR, MLR, SII, SIRI, and AISI, are increasingly recognized as accessible indicators of immune activity and tumor burden in oncology [11,17].

In recent years, there have been increased efforts to better understand the relationship between inflammatory markers and their impact on disease severity, clinical outcomes, and therapeutic regimens. Integrating these findings into the clinical routine can be crucial in providing patients with more effective treatment regimens [18]. Additionally, in the era of modern medicine, a personalized approach for each patient, considering both clinical and paraclinical characteristics, remains a crucial aspect to be considered.

Lung cancer is broadly categorized into two major histologic types: NSCLC and SCLC. Furthermore, NSCLC is classified mainly into three histologic subtypes: adenocarcinoma, squamous cell carcinoma, and large cell carcinoma [19]. While the role of inflammation in cancer is well established, its variation across histologic subtypes of lung cancer remains underexplored. Therefore, the impact of histologic findings in lung cancer patients and their associated response to therapeutic regimens needs to be considered and integrated as an essential part of the clinical evaluation and management of these high-risk patients. To date, few studies have examined the impact of histology on the inflammatory response.

Therefore, this study aimed to represent one of the first steps toward a deeper understanding of the complex relationship between inflammation, histology, and disease severity in lung cancer patients. The ultimate goal was to highlight key findings that may guide therapeutic approaches and optimize the overall management of lung cancer patients.

## 2. Materials and Methods

### 2.1. Data Sources and Patients Included in the Study

A retrospective descriptive study of 202 patients diagnosed with lung carcinoma between 1 January 2019 and 31 December 2023, at the Clinical County Hospital Mures, Târgu Mureș, Romania, was conducted. The study was conducted following the Declaration of Helsinki and was approved by the Ethics Committee of the Clinical County Hospital Mureș (approval number 20419, dated 15 December 2023).

Inclusion and exclusion criteria were applied to select the proper study group.

Patients aged ≥18 years with histologically confirmed lung carcinoma and complete CBC data at diagnosis were included. Those with active infections or other malignancies were excluded.

The analyzed parameters included the following:

1. Tumor-related parameters:
  The histological type of lung carcinoma: NSCLC (adenocarcinoma, squamous cell carcinoma, adenosquamous carcinoma, and NSCLC not-otherwise-specified (NOS)) and SCLC.
  The stage of the tumor at diagnosis was based on the TNM classification of malignant tumors, where T describes the primary tumor size and site, N represents the involvement of the regional lymph nodes, and M represents the presence of distant metastasis.
2. Laboratory parameters:
  For all included patients, whole-blood venous samples were collected in the morning, after an overnight fast, and analyzed with a Mindray BC-6200 automatic hematology analyzer (Mindray Medical International Limited, Shenzhen, China).
  - Parameters derived from the CBC of the patients at the time of initial diagnosis: leukocyte count, neutrophil count, lymphocyte count, monocyte count, platelet count, and eosinophil count.
  - The following CBC-derived inflammatory indexes: eosinophil-to-neutrophil ratio (ENR); neutrophil-to-lymphocyte ratio (NLR); derived neutrophil-to-lymphocyte ratio (d-NLR); eosinophil-to-monocyte ratio (EMR); monocyte-to-lymphocyte ratio (MLR); platelet-to-lymphocyte ratio (PLR); systemic inflammatory response index (SIRI), aggregate index of systemic inflammation (AISI), and systemic inflammatory index (SII).

The formulas used to calculate the CBC-derived indexes are presented in Table 1.

3. Demographic and general parameters:
  - General data regarding the gender and the age of the patients at diagnosis, the living environment (urban/rural), the body mass index (BMI), smoking status, and COPD as comorbidities.
  - BMI was calculated using the following formula: BMI = kg/m^2^. Based on BMI, patients were classified as underweight (BMI < 18.5 kg/m^2^), normal weight (BMI between 18.5 and 24.99 kg/m^2^), overweight (BMI between 25 and 29.99 kg/m^2^), grade I obesity (BMI between 30 and 34.99 kg/m^2^), grade II obesity (BMI between 35 and 39.99 kg/m^2^), grade III obesity (BMI > 40 kg/m^2^).

### 2.2. Statistical Analysis of Data

The statistical analysis was performed using MedCalc Statistic software for Windows, version 23.0.2. Normality was tested using the Shapiro–Wilk test. Continuous variables were expressed as the median or mean and standard deviation, while categorical variables were given the absolute count (n) and proportions. The Chi-square test compared categorical variables, while the independent Mann–Whitney test was used for continuous variables. Logarithmic ANOVA was used to compare groups, followed by a post hoc Dunn–Bonferroni test when applicable. A *p*-value of 0.05 was considered statistically significant.

## 3. Results

### 3.1. General Characteristics of the Study Population

Table 2 lists the main characteristics of the included patients, including data about demographic parameters such as BMI distribution, smoking status, and associated comorbidities.

### 3.2. Histological Types of Lung Carcinoma and Stage at Diagnosis

In our study group, 27 patients were diagnosed with SCLC and 175 with NSCLC.

**Figure 1:**
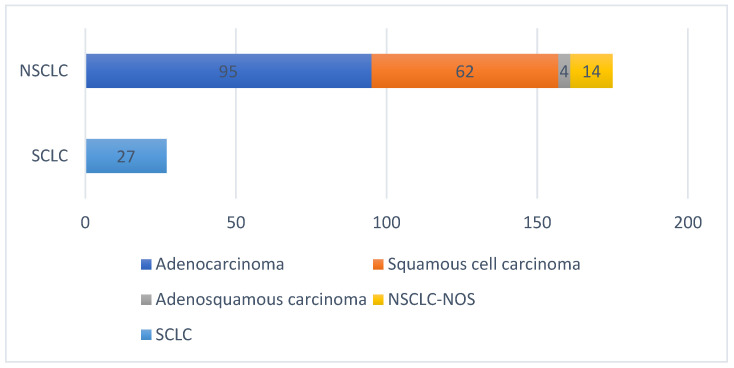
Histologic type of lung cancer of the included patients.

In the NSCLC group, 95 patients were diagnosed with adenocarcinoma, 62 with squamous carcinoma, 14 with Not-Otherwise-Specified NSCLC (NOS-NSCLC), and 4 with adenosquamous lung carcinoma, as shown in Figure 1.

**Figure 2:**
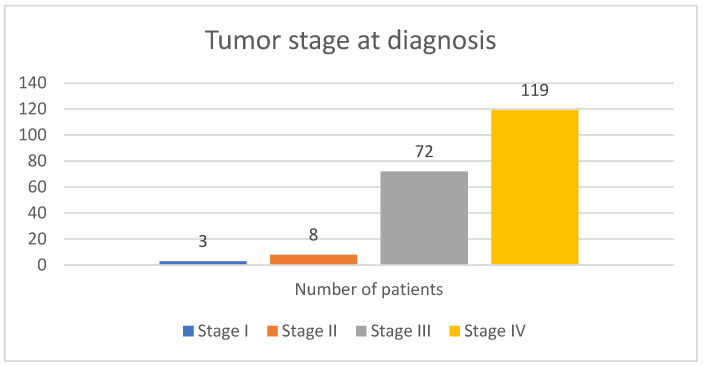
Tumor stage at diagnosis.

Regarding stage at diagnosis, most patients were diagnosed in the advanced stages of the disease (stages III and IV). Three patients were diagnosed with stage I, 8 were diagnosed with stage II, 72 were diagnosed with stage III, and 119 were diagnosed with stage IV. Figure 2 presents the tumor stage at diagnosis for the included study population.

Additionally, Table 3 presents detailed data on the relationship between histological type and tumor stage at the initial diagnosis.

### 3.3. Assessment of the Inflammatory Response in Lung Cancer Patients

The first step of the study was to compare the inflammatory response encountered in our study population with the reference values cited in the literature. The reference values used were as follows: Leukocytes ×10^3^/µL: 4.1–12.2; Neutrophils ×10^3^/µL: 1.50–7.90; Lymphocytes ×10^3^/µL: 1.10–3.40; Monocytes ×10^3^/µL: 0.30–1.10; Eosinophils ×10^3^/µL: 0.00–0.50; Platelets ×10^3^/µL: 150–400; NLR: 0.78–3.53 (13); d-NLR: ≤2.2; MLR: 0.352–0.369; PLR: <185; EMR: 0.02–0.24; ENR: <0.05; SII: 660 (NSCLC)/1600 (SCLC); AISI: 351; SIRI: 2;

We analyzed these aspects, as shown in Table 4, to better visualize and understand the impact of disease stage and histologic type on the inflammatory response.

Our results showed that the CBC-derived inflammatory markers were within normal ranges in stage I for all the histologic subtypes of lung cancer. For patients presenting with stage II disease at diagnosis, the inflammatory response was within normal ranges for adenosquamous, non-small cell lung cancer (NSCLC-NOS), and small cell lung cancer (SCLC). In contrast, adenocarcinoma and squamous cell carcinoma patients presented with six elevated parameters, as can be seen in Table 4.

CBC-derived inflammatory markers progressively increased with tumor stage in both NSCLC and SCLC, with NSCLC-NOS showing particularly high values, suggesting a more aggressive inflammatory tumor environment. Seven parameters were elevated in stage III and stage IV patients: NLR, d-NLR, PLR, MLR, SII, AISI, and SIRI for both adenocarcinoma and squamous cell carcinoma patients. In contrast, the inflammatory response varied among patients with adenosquamous, NSCLC-NOS, and SCLC.

An additional finding was that the lymphocyte count, although within normal ranges, tended to decrease as disease severity progressed in all histologic subtypes of lung cancer, except for NSCLC-NOS, where it increased in stage IV patients.

### 3.4. Analysis of the Inflammatory Response Variations in Lung Cancer Patients Based on Histology

The next step in the study was to determine if there were any differences in the inflammatory response of lung cancer patients based solely on histological classification.

For this purpose, we initially compared the inflammatory response encountered in NSCLC and SCLC patients. Afterward, we studied and compared the inflammatory response variations encountered in the histologic subtypes of NSCLC: adenocarcinoma, squamous cell carcinoma, adenosquamous carcinoma, and NSCLC-NOS.

#### 3.4.1. Comparison Between the Inflammatory Response in NSCLC Versus SCLC Patients

The inflammatory status was assessed by analyzing the levels of CBC-derived parameters mentioned in Table 1 at the time of initial diagnosis and the counts of leukocytes, neutrophils, lymphocytes, monocytes, and eosinophils. Table 5 presents detailed information regarding the analyzed parameters and the main differences between NSCLC and SCLC patients.

Our study reveals that, although within normal ranges, all cellular lines, except platelet count, were more elevated in NSCLC patients than in those with SCLC. In addition, regarding the CBC-derived inflammatory indexes, NLR, d-NLR, PLR, AISI, and SIRI were elevated in both NSCLC and SCLC patients. In contrast, MLR and SII were elevated in NSCLC patients only.

As shown in Table 5, no statistically significant difference was found between the inflammatory responses of patients with NSCLC and those with SCLC.

#### 3.4.2. Comparison Between the Inflammatory Response in Different Histological Subtypes of NSCLC: Adenocarcinoma Versus Squamous Cell Carcinoma Versus NOS Versus Adenosquamous Carcinoma

The second step of the study was to test whether the histologic subtype of NSCLC influences the inflammatory response. As previously discussed, Table 4 presents the inflammatory changes encountered in the various NSCLC subtypes.

Additionally, we searched for parameters that differed statistically across the various histologic subtypes of NSCLC.

**Figure 3:**
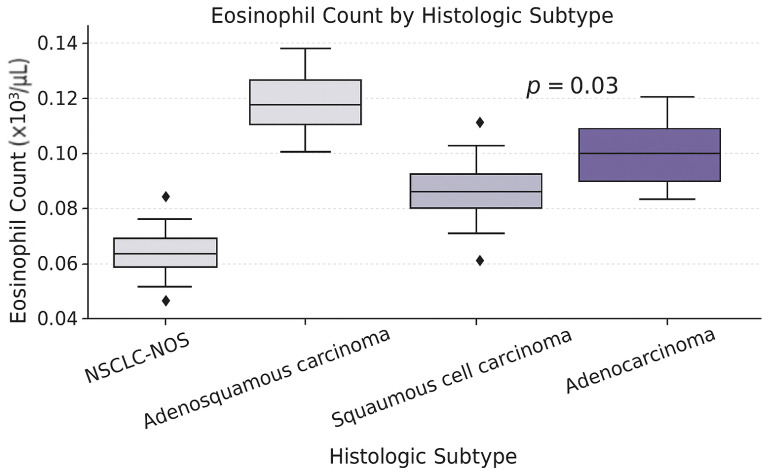
Eosinophil count in NSCLC histologic subtypes.

Our results showed that eosinophil counts differed significantly among NSCLC subtypes (*p* = 0.003). They were significantly higher in patients with adenosquamous carcinoma and adenocarcinoma than in those with NSCLC-NOS, as shown in Figure 3.

### 3.5. Analysis of Disease Severity Impact on the Inflammatory Response in Lung Cancer Patients

This part of the study aimed to identify and compare the influence of disease severity, as classified by the TNM system, on distinct CBC-derived inflammatory biomarkers in patients diagnosed with the same histologic subtype of lung cancer. For this purpose, we examined the common and particular aspects of the inflammatory response in patients diagnosed with different stages of the disease and the same histological subtype.

The analysis was performed for patients with SCLC and NSCLC (adenocarcinoma, squamous cell carcinoma, adenosquamous carcinoma, and NSCLC-NOS).

**Figure 4:**
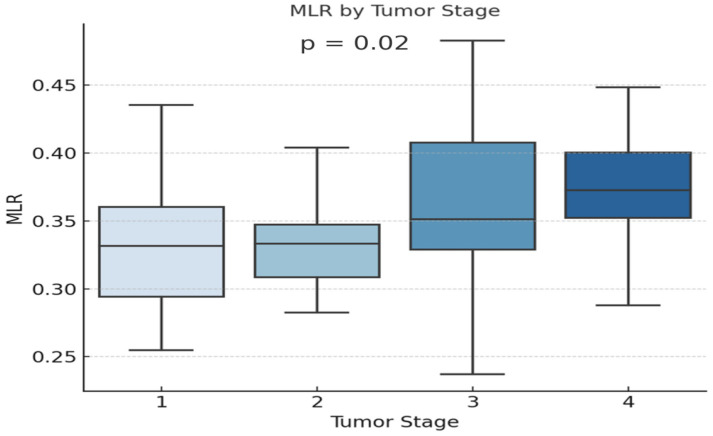
Evolution of MLR levels in different stages of severity in adenocarcinoma patients.

Our results showed that MLR increased significantly with tumor stage in adenocarcinoma patients (*p* = 0.02), as shown in Figure 4.

**Figure 5:**
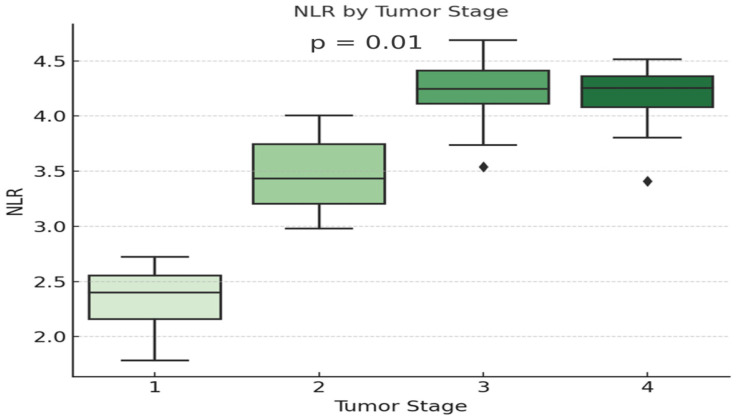
Evolution of NLR levels in different stages of severity in adenocarcinoma patients.

The same trend was observed for NLR levels, which increased significantly with tumor stage in adenocarcinoma patients (*p* = 0.01), as seen in Figure 5.

Although no other statistically significant findings were observed regarding the impact of tumor stage at diagnosis on the inflammatory response in other histologic subtypes of lung cancer, our findings provide meaningful and relevant scientific data regarding the complex variability of inflammation in different histologic subtypes of lung cancer.

For adenocarcinoma patients, leukocytes, neutrophils, lymphocytes, and monocytes had the highest counts in stage II, similar to AISI and SIRI levels. In contrast, PLR and EMR had the lowest values in patients with stage II adenocarcinoma.

A similar trend was observed in squamous cell carcinoma patients, as the counts of leukocytes, neutrophils, lymphocytes, and eosinophils, similar to EMR and ENR, exhibited the highest counts in stage II patients. In contrast, for squamous cell carcinoma patients, NLR, d-NLR, MLR, PLR, SII, and AISI exhibited a progressive increase as the tumor stage progressed, with the highest values observed in more advanced stages (III and IV).

An interesting finding was observed in adenosquamous carcinoma patients, where all cellular lines exhibited higher levels in stage III patients compared to stage IV. In contrast, all CBC-derived inflammatory indexes, except for EMR, ENR, and AISI, were higher in stage IV patients than in stage III patients.

For NSCLC-NOS patients, we observed a significant decrease in platelet count, SII, AISI, and SIRI levels in stage IV patients compared to stage III patients, as Table 6 depicts.

For SCLC patients, leukocyte, neutrophil, lymphocyte, and platelet counts were higher in stage III patients than in stage IV. In contrast, regarding the CBC-derived inflammatory markers, our results showed that NLR, EMR, and SIRI exhibited increased levels in stage III compared to stage IV patients. In contrast, d-NLR, MLR, PLR, ENR, SII, and AISI were more elevated in stage IV patients than in stage III.

### 3.6. Analysis of the Combined Impact of Histology and Tumor Stage on the Inflammatory Response in Lung Cancer Patients

#### Comparison Between Different Stages of Different Histological Subtypes

The final step of the study was to determine the variation in the inflammatory response according to the stage and the histological type of the tumor. No statistically significant difference was found in stages I and II of the inflammatory response. However, in stage III, ENR (*p* = 0.002), EMR (*p* = 0.006), AISI (*p* = 0.017), eosinophil count (*p* < 0.001), and platelet count (*p* = 0.022) differed significantly across different histological subtypes.

Adenosquamous patients presented the highest levels of ENR (0.11 [0.09–0.14]) while NOS patients presented the lowest levels (0.003 [0.002–0.004]). EMR levels were the highest in SCLC (0.24 [0.16–0.29]) and squamous cell carcinoma patients (0.22 [0.19–0.26]) and the lowest in NSCLC-NOS patients (0.05 [0.04–0.07]). Eosinophil count was significantly higher in adenosquamous patients (1.13 [1.05–1.19]) versus NSCLC-NOS patients (0.04 [0.03–0.05]) while platelet count was the highest in NOS patients (810 [798.2–824.5]) compared to squamous carcinoma patients (323.6 [286.48–362.81]), where it exhibited the lowest levels. AISI (*p* = 0.017) was found to be the highest in NSCLC-NOS patients (5407.92 [5403.2–5414.6]) and the weakest in SCLC patients (840.30 [719–1248.4]).

In stage IV patients, AISI (*p* = 0.032), SIRI (*p* = 0.036), and SII (*p* = 0.041) varied significantly between histological subtypes. AISI (1205.18 ± 1376.25) and SII (1834.38 ± 1568.81) had the highest levels in adenosquamous carcinoma patients and the lowest levels in adenocarcinoma patients (879.584 [555.66–1169.23] and 1299.42 [1097.37–1946.97], respectively). In addition, SIRI was the highest in NSCLC-NOS (3.54 [2.73–8.15]) and adenosquamous carcinoma patients (3.54 ± 3.44) and the lowest in SCLC patients (2.66 [1.63–3.38])

Table 7 presents the relationship between the CBC-derived parameters, which differ based on histology and disease severity.

## 4. Discussion

Complex pathological pathways are involved in the inflammatory response associated with malignancies, encompassing angiogenesis, immunosuppression, genetic instability, tumor progression, and metastasis capacity [19]. Chronic inflammation leads to necrosis of healthy cells due to malignant cells, as well as to DNA damage, reactive oxygen species (ROS) accumulation, and alterations in signaling pathways, including p53 and K-RAS [20,21,22].

Our results showed that disease severity impacts the associated inflammatory response in all histologic subtypes of lung cancer to varying degrees, depending on the stage of severity. For stage I patients, all CBC-derived inflammatory biomarkers were within normal ranges. As tumor severity progressed, the inflammatory response also intensified. The histologic subtype of the tumor influences the associated inflammatory response, as evidenced by the variation in elevated systemic inflammatory markers between SCLC and NSCLC patients, as well as between different histologic subtypes of NSCLC, findings supported by previous research conducted by Rice and Belani [23]. The inflammatory response encountered in our study population was generally less pronounced in SCLC patients than in those with NSCLC, despite SCLC patients having a decreased survival rate and quality of life compared to those with NSCLC. Previous studies support our results, as SCLC patients tended to have lower levels of systemic inflammation than NSCLC patients [23].

When observing NSCLC and its histologic subtypes, the results showed that patients with adenocarcinoma and squamous cell carcinoma shared major common pathways in their inflammatory responses. In contrast, patients with other NSCLC subtypes, such as NSCLC-NOS and adenosquamous carcinoma, exhibited both shared and distinct pathways in their inflammatory responses. In advanced stages, patients diagnosed with NSCLC-NOS exhibited the most numerous CBC-derived inflammatory markers outside the normal range, suggesting that inflammation might be an essential part in NOS patients’ evolution and prognosis, as a histologic diagnosis of NOS may indicate poor differentiation of tumor cells [24]. Future studies are needed to gain a deeper understanding of the highly altered inflammatory response observed in NSCLC-NOS patients, ultimately leading to a more comprehensive understanding of the complex pathophysiological mechanisms underlying this histologic subtype of lung cancer.

A crucial aspect of the carcinogenesis process is associated with the presence of white blood cells (WBCs), as they reflect the body’s immune and inflammatory responses and are thought to promote carcinogenesis and angiogenesis, either directly or indirectly, by stimulating cytokine-driven pathways [25,26,27]. Additionally, the number of leukocytes, neutrophils, platelets, and lymphocytes fluctuates during tumoral growth and lysis due to the associated secretion of cytokines [28,29].

In the current study, when observing the evolutionary trend in cellular lines, we noted that, although within normal ranges, the lymphocyte count highly decreased in stage IV patients compared to less severe stages in all histologic subtypes of lung cancer, except for NSCLC-NOS. This might be explained by suppressing lymphocytes during metastatic processes as the disease progresses [30]. Additionally, lymphopenia may facilitate tumor development and growth, thereby promoting carcinogenesis [31].

Eosinophils have emerged as promising cells impacting carcinogenesis and have gained interest for their dual role in cancer, exhibiting both immunosuppressive and immune-activating effects [32]. Studies have found that moderate pre-treatment eosinophil levels (100–500 cells/μL) are associated with better overall survival (OS) in advanced NSCLC patients [33], and high eosinophil counts correlate with improved treatment response and OS in late-stage NSCLC [34,35,36].

Our study revealed that stage III adenosquamous carcinoma patients had elevated eosinophil counts and ENR and EMR levels. In addition, as presented in Section 3.4.2, the eosinophil count was found to be the only parameter exhibiting significant statistical variations across the histologic subtypes of NSCLC patients, with the highest values being observed in patients with adenosquamous and adenocarcinoma and the lowest in those with NSCLC-NOS. Long et al. also addressed the relationship between high eosinophil count and the risk of developing carcinoma, citing high eosinophil levels as an additional risk factor for developing adenocarcinoma [37].

Platelets play a critical role in tumor progression by promoting metastasis, angiogenesis, and microthrombotic events [38]. Malignant cells can activate and aggregate platelets, enhancing tumor growth and cancer-related inflammation [39,40,41]. Our findings revealed significant differences in platelet counts among stage III lung cancer patients based on histologic subtype. Notably, patients with NSCLC-NOS exhibited the highest platelet levels, suggesting a heightened risk of platelet-mediated inflammatory responses. In contrast, those with squamous cell carcinoma had the lowest counts. Elevated platelet levels have previously been linked to increased metastasis and disease progression [28,42].

Monocytes, a key white blood cell subpopulation, have a dual role in lung cancer, acting either protectively or as facilitators of tumor progression depending on the subtype [37]. Prior studies have associated high monocyte counts with poorer survival and increased recurrence risk in advanced lung cancer [43,44]. In our research, monocyte-derived inflammatory markers, particularly the monocyte-to-lymphocyte ratio (MLR), were elevated in advanced stages (III and IV) of both SCLC and NSCLC, across all histologic subtypes. In adenocarcinoma patients, MLR was significantly correlated with disease stage (*p* = 0.01), increasing progressively. Thrombocytosis has been associated with poor overall survival in lung cancer patients and is considered an independent negative prognostic factor [39]. Therefore, integrating these easily accessible hematologic markers into the routine clinical assessment of patients could help in the stratification, prognosis, and personalized treatment planning in lung cancer management.

The neutrophil-to-lymphocyte ratio (NLR), a well-established prognostic marker in various malignancies, including lung cancer [45,46], is typically elevated due to increased neutrophils or decreased lymphocytes, both common in cancer-related immunosuppression and tumor progression [47,48]. In our cohort, NLR levels were significantly correlated with tumor stage only in adenocarcinoma patients, reinforcing its prognostic value in this subtype. No such correlation was found in other NSCLC or SCLC, suggesting that histologic subtype may significantly influence inflammatory responses. This may be attributed to differences in the tumor microenvironment (TME) and the cytokine-secreting capabilities of cancer cells, which can vary by histology [49] or can be explained by the lower number of patients diagnosed with NSCLC-NOS, NSCLC-Adenosquamous carcinoma, and SCLC. Further studies are needed to understand and assess the primary differences between local and systemic inflammation, as depicted in the various histologic subtypes of SCLC and NSCLC.

As more and more scientific studies propose inflammation as a key factor in tumor progression, novel complex CBC-derived inflammatory indexes are also suggested as prognostic factors for cancer patients. The systemic immune–inflammation index (SII) was first mentioned in 2014 by Hu et al. as a novel marker of inflammation [50]. A study published by Mazzella et al. [51] cited SII as a prognostic factor for resected lung cancer, while Fournel et al. [52] linked the prognosis of patients diagnosed with malignant pleural mesothelioma with the NLR and SII values. The same results were confirmed by Chen et al., who revealed that high SII was a significant prognostic factor for NSCLC patients [45]. The systemic inflammation response index (SIRI) can predict survival in various malignancies, such as oral squamous cell carcinoma and pancreatic cancer [53]. A study by Liu et al. stated that high SIRI levels could be linked with a decreased survival rate in surgically treated NSCLC patients [26]. In contrast, other studies did not identify a relationship between SIRI and the survival duration of NSCLC patients [54]. Increased levels of AISI and SIRI were correlated in a study published by Feier et al. with higher hospital stay rates and postoperative complications [55].

Our study corroborates these findings and highlights AISI as the most discriminative marker in stage III lung cancer, showing the highest levels in NSCLC-NOS patients. In stage IV disease, AISI, SIRI, and SII varied significantly across histological subtypes, with the highest levels observed in patients with adenosquamous carcinoma. These results suggest that these indices, particularly in advanced stages, may reflect histology-dependent variations in systemic inflammatory response and could assist in prognostication and stratifying patients for tailored monitoring or treatment intensification. Further investigation is warranted to clarify the inflammatory profiles specific to less common subtypes, such as NSCLC-NOS and adenosquamous carcinoma.

In the final part of our analysis, we assessed lifestyle factors and main comorbidities. COPD was present in 40.1% of patients, and smoking was present in 86.6% of the included patients. This strong co-occurrence is clinically relevant, as smoking and COPD increase mortality in lung cancer patients due to the pro-inflammatory and carcinogenic effects of tobacco-derived compounds such as peroxides and nitrosamines [19,56,57]. In addition, low hemoglobin levels were identified in 71.3% of our cohort (144/202 patients), at a higher rate than the one reported in the literature (38%). Pre-treatment anemia is known to be linked to poor prognosis and survival in cancer patients [58]. Therefore, our findings reinforce the importance of recognizing and addressing these associated characteristics in the comprehensive management of lung cancer patients.

Our study confirmed differences in the inflammatory response across various histologic subtypes of lung cancer, aiming to be one of the first steps toward a better understanding of the complexity of immune interactions between different cellular lines, CBC-derived inflammatory parameters, and lung cancer histology. The study aims to present significant clinical findings that can be implemented into routine clinical practice. Based on the analysis of the CBC-derived inflammatory profiles at the time of initial diagnosis, risk stratification could be easily performed for all patients, leading to a closer follow-up and an associated multidisciplinary approach. In addition, data about the histologic subtype of lung cancer could help in the early identification of those patients at higher risk of increased severity and improve the treatment personalization and supportive care.

The current research has several limitations, including its retrospective perspective and the need to extend and integrate its results into clinical protocols. We consider that a critical perspective from which lung cancer patients would greatly benefit would be the identification of quickly accessible CBC-derived inflammatory biomarkers for each histological subtype of lung cancer. Therefore, identifying high-risk patients at the time of initial diagnosis based on the aspect mentioned above could guide treatment regimens, personalize therapeutic options, and lead to better outcomes for this highly deadly condition.

The present study has shown that a deeper understanding of the complex concept of tumor-associated inflammation, in concordance with the tumor’s histologic subtype, leads to an improved quality of life for cancer patients and an increased overall survival rate.

## 5. Conclusions

Our findings address the critical importance of tailoring anti-inflammatory strategies to the histologic subtype of lung cancer, reinforcing the role of inflammation as a central component in tumor progression and prognosis. CBC-derived inflammatory markers vary by histologic subtype and may help guide early risk assessment and personalized treatment planning. Future work will aim to validate these findings in prospective studies and add relevant information to the prognostic role of systemic inflammation, such as associated sarcopenic changes. We intend to strengthen the results of this study by defining and integrating into the complex assessment of lung cancer patients of a new prognostic score, which incorporates four main characteristics of lung cancer patients: histology, TNM stage, and inflammatory and sarcopenic changes, with the final goal to guide personalized treatment regimens and improve the overall survival of lung cancer patients.

## Figures and Tables

**Table 1:** Formulas for Calculating CBC-Derived Inflammatory Indexes.

Parameter	Formula
Neutrophil-to-lymphocyte ratio (NLR)	Neutrophil count/lymphocyte count [×10^3^/μL]
Derived neutrophil-to-lymphocyte ratio (d-NLR)	Neutrophil count/(WBC − neutrophil count) [×10^3^/μL]
Monocyte-to-lymphocyte ratio (MLR)	Monocyte count/lymphocyte count [×10^3^/μL]
Platelet-to-lymphocyte ratio (PLR)	Platelet count/lymphocyte count [×10^3^/μL]
Eosinophil-to-neutrophil ratio (ENR)	Eosinophil count/neutrophil count [×10^3^/μL]
Eosinophil-to-monocyte ratio (EMR)	Eosinophil count/monocyte count [×10^3^/μL]
Systemic Inflammatory Index (SII)	(Neutrophil count × platelet count)/lymphocyte count [×10^3^/μL]
Systemic Inflammatory Response Index (SIRI)	(Neutrophil count × monocyte count)/lymphocyte count [×10^3^/μL]
Aggregate index of systemic inflammation (AISI)	(Neutrophil count × monocyte count × platelet count)/lymphocyte count [×10^3^/μL]

**Table 2:** General characteristics of the study population.

Parameters	N (Absolute Count)	N (Percentage %)
	ALL N = 202 patients	
	AGE—mean: 66.62 ± 8.34	
<50 years	4	1.98%
50–59 years	37	18.32%
60–69 years	83	41.09%
70–79 years	71	35.15%
≥80 years	7	3.46%
	Gender	
MALE	150	74.25%
FEMALE	52	25.75%
	Living environment	
RURAL	118	58.42%
URBAN	84	41.58%
BMI—a median of numeric values (when available): 24 [23.328–24.653]		
<18.5	36	17.82%
18.5–24.99	105	51.98%
25–29.99	39	19.30%
30–34.99	14	6.94%
35–39.99	6	2.97%
>40	2	0.99%
	Associated COPD	
YES	81	40.1%
NO	121	59.9%
	Hb levels	
Normal Hb levels	58	28.71%
Low Hb levels	144	71.29%
	Smoking	
YES	175	86.63%
NO	27	13.37%

**Table 3:** Distribution of Lung Cancer Histologic Subtypes by Tumor Stage at Diagnosis.

Histopathological Subtype	Stage I	Stage II	Stage III	Stage IV
NSCLC—Adenocarcinoma	2	4	23	66
NSCLC—Squamous cell carcinoma	1	4	39	18
NSCLC—Adenosquamous carcinoma	-	-	2	2
NSCLC—NOS	-	-	1	13
SCLC	-	-	7	20

**Table 4:** Elevated CBC-Derived Inflammatory Markers by Subtype and Stage.

Elevated Levels of CBC-Derived Inflammatory Parameters Based on Histologic Subtype and Tumor Stage at Diagnosis				
*NSCLC* *—Adenocarcinoma*	*NSCLC* *—Squamous Cell Carcinoma*	*NSCLC—Adenosquamous Carcinoma*	*NSCLC—NOS*	*SCLC*
***Stage I*** ○ None	***Stage I*** ○ None	***Stage I*** ○ None	***Stage I*** ○ None	***Stage I*** ○ None
***Stage II*** ○ Neutrophils ×10^3^/µL—8.53 [7.9–11.27] ○ NLR—3.51 [3.42–3.73] ○ d-NLR—2.23 [2.1–2.65] ○ SII—843.6 [840.3–848.2] ○ AISI—964.21 [963.8–964.9] ○ SIRI—3.32 [3.21–3.49]	***Stage II*** ○ Neutrophils ×10^3^/µL—8.19 [7.8–8.53] ○ NLR—3.92 [3.82–4.06] ○ d-NLR—2.50 [2.32–2.93] ○ SII—1253.62 [1251.4–1257.3] ○ AISI—865.47 [861.2–872.3] ○ SIRI—2.67 [2.58–2.76]	***Stage II*** ○ None	***Stage II*** ○ None	***Stage II*** ○ None
***Stage III*** ○ NLR—4.12 [3.70–5.07] ○ d-NLR—2.62 [2.32–2.95] ○ MLR—0.40 [0.30–0.45] ○ PLR—222.90 [176.55–270.70] ○ SII—1545.91 [1100.19–2003.99] ○ AISI—925.56 [697.95–1159.28] ○ SIRI—2.52 [2.02–3.49]	***Stage III*** ○ NLR—4.12 [3.70–5.07] ○ d-NLR—2.62 [2.32–2.95] ○ MLR—0.40 [0.30–0.45] ○ PLR—222.90 [176.55–270.70] ○ SII—1545.91 [1100.19–2003.99] ○ AISI—925.56 [697.95–1159.28] ○ SIRI—2.52 [2.02–3.49]	***Stage III*** ○ Eosinophils ×10^3^/µL—1.11 [1.05–1.19] ○ Platelets ×10^3^/µL—430 ± 250.31 ○ PLR—205.20 ±166.40 ○ EMR—1.13 [1.08–1.19] ○ ENR—0.11 [0.09–0.14] ○ SII—1809.11 ± 1920.87 ○ AISI—1741.6 ± 2105.94 ○ SIRI—3.16 ± 3.06	***Stage III*** ○ Leukocytes ×10^3^/µL—12.99 ± 6.3 ○ Neutrophils ×10^3^/µL—10.73 ± 5.1 ○ Platelets ×10^3^/µL—810 [798.2–824.5] ○ NLR—7.95 [7.85–8.19] ○ d-NLR—4.75 [4.65–4.87] ○ MLR—0.62 [0.56–0.71] ○ PLR—600 [595.3–607.3] ○ SII—6438 [6021.2–6743.3] ○ AISI—5407.92 [5403.2–5414.6] ○ SIRI—6.676 [6.58–6.72]	***Stage III*** ○ Platelets ×10^3^/µL—348.96 ± 149.16 ○ NLR—4.4 [3.9–5.9] ○ d-NLR—2.99 ± 1.63 ○ PLR—187.16 [171.72–311] ○ AISI—840.30 [719–1248.4] ○ SIRI—2.72 [1.63–3.28]
***Stage IV*** ○ NLR—5.27 [3.42–6.14] ○ d-NLR—3.07 [2.40–3.77] ○ MLR—0.39 [0.30–0.55] ○ PLR—249.75 [168.24–325.23] ○ SII—1687.62 [1236.83–2190.83] ○ AISI—985.08 [776.30–1429.09] ○ SIRI—2.67 [2.15–4.17]	***Stage IV*** ○ NLR—5.27 [3.42–6.14] ○ d-NLR—3.07 [2.40–3.77] ○ MLR—0.39 [0.30–0.55] ○ PLR—249.75 [168.24–325.23] ○ SII—1687.62 [1236.83–2190.83] ○ AISI—985.08 [776.30–1429.09] ○ SIRI—2.67 [2.15–4.17]	***Stage IV*** ○ NLR—5.76 ± 3.24 ○ d-NLR—3.31± 0.90 ○ PLR—317.39 ± 221.50 ○ SII—1834.38 ± 1568.81 ○ AISI—1205.18 ± 1376.25 ○ SIRI—3.54 ± 3.44	***Stage IV*** ○ Leukocytes ×10^3^/µL—12.80± 6.01 ○ Neutrophils ×10^3^/µL—10.35 ± 6.03 ○ NLR—5.54 [4.29–8.17] ○ d-NLR—3.43 [2.79–5.71] ○ MLR—0.54 [0.33–0.79] ○ PLR—204.68 [170.54–253.13] ○ SII—1861.48 [1470.32–2701.95] ○ AISI—1191.34 [776.81–3329.93] ○ SIRI—3.546 [2.73–8.15]	***Stage IV*** ○ Platelets ×10^3^/µL—339.7 ± 146.86 ○ NLR—4.36 [3.28–6.22] ○ d-NLR—3.12 ± 1.4 ○ MLR—0.39 [0.27–0.46] ○ PLR—193.58 [126.21–320.88] ○ AISI—886.82 [42.35–1297.4] ○ SIRI—2.66 [1.63–3.38]

**Table 5:** CBC-Derived Inflammatory Biomarkers: NSCLC versus. SCLC.

Parameter	NSCLC	SCLC	*p*-Value
Leukocytes ×10^3^/µL	10 [9.25–10.76]	9.59 [6.75–10.52]	0.2022
Neutrophils ×10^3^/µL	7.37 [6.46–7.94]	6.49 [5.18–9.04]	0.5935
Lymphocytes ×10^3^/µL	1.71 [1.5–1.79]	1.59 [1.18–1.9]	0.9966
Monocytes ×10^3^/µL	0.62 [0.59–0.65]	0.61 ± 0.23	0.2698
Eosinophils ×10^3^/µL	0.12 [0.11–0.14]	0.11 [0.08–0.17]	0.9826
Platelets ×10^3^/µL	325 [305.06–352.56]	348.96 ± 149.16	0.7250
NLR	4.35 [3.98–5.02]	4.4 [3.89–5.88]	0.1205
d-NLR	2.84 [2.53–3.05]	2.99 ± 1.63	0.2645
MLR	0.38 [0.35–0.42]	0.36 [0.32–0.45]	0.0684
PLR	204.69 [180.49–220.63]	187.16 [171.72–311.6]	0.2940
EMR	0.2 [0.16–0.23]	0.23 [0.16–0.29]	0.7580
ENR	0.02 [0.01–0.02]	0.016 [0.012–0.02]	0.4277
SII	1591.62 [1304.44–1811.56]	1430.94 [1033–1903.68]	0.2511
AISI	952.25 [804.12–1105.3]	840.3 [719–1248.4]	0.0739
SIRI	2.77 [2.4–2.96]	2.72 [1.63–3.28]	0.0762

The values in the square brackets represent the measure of dispersion of the inflammatory biomarkers in the study population.

**Table 6:** Variations in the inflammatory biomarkers in stage III and IV NSCLC-NOS patients.

Inflammatory Parameter	Stage III	Stage IV
Platelets count	810 × 10^3^/µL	344 × 10^3^/µL
SII	6438	1861.48
AISI	5407.92	1191.34
SIRI	6.676	3.546

**Table 7:** Summary of Inflammatory Markers Differing by Histologic Subtype and Tumor Stage.

Stage	Parameter	*p*-Value
I	None	-
II	None	-
III	AISI	*p* = 0.017
	ENR	*p* = 0.002
	EMR	*p* = 0.006
	Eosinophil count	*p* < 0.001
	Platelets count	*p* = 0.022
IV	AISI	*p* = 0.022
	SIRI	*p* = 0.022
	SII	*p* = 0.041

## Data Availability

No new data were created or analyzed in this study. Data sharing is not applicable to this article.

## Abbreviations

CBC	Complete blood count
NLR	Neutrophil-to-lymphocyte ratio
d-NLR	Derived neutrophil-to-lymphocyte ratio
MLR	Monocyte-to-lymphocyte ratio
PLR	Platelet-to-lymphocyte ratio
ENR	Eosinophil-to-neutrophil ratio
EMR	Eosinophil-to-monocyte ratio
SII	Systemic inflammatory index
SIRI	Systemic Inflammatory Response Index
AISI	Aggregate index of systemic inflammation
SCLC	Small cell lung carcinoma
NSCLC	Non-small cell lung carcinoma
WHO	World Health Organization
TME	Tumor microenvironment
CAFs	Cancer-associated fibroblasts
NOS	Not-otherwise-specified carcinoma
TNM	Tumor, Node, Metastasis system
BMI	Body mass index
COPD	Chronic obstructive pulmonary disorder
Hb	Hemoglobin
ROS	Reactive oxygen species
KRAS	Kirsten Rat Sarcoma viral oncogene
WBCs	White blood cells
OS	Overall survival
MRI	Magnetic Resonance Imaging
CT	Computed Tomography
